# Synergistic Effects of Amino Acids and *Bacillus velezensis* N35 on Suppressing *Phelipanche aegyptiaca* Parasitism and Modulating Tomato Growth: Insights from Transcriptomic Profiling

**DOI:** 10.3390/plants15091327

**Published:** 2026-04-27

**Authors:** Wei He, Yiguang Wang, Siqiong Tang, Wenfang Luo, Xin Huang, Junhui Zhou, Xiang Zhang, Jianjun Xu

**Affiliations:** 1 Institute of Plant Protection, Academy of Agricultural Sciences of Xinjiang Uygur Autonomous Region, Key Laboratory of Integrated Pest Management on Crops in Northwestern Oasis, Ministry of Agriculture and Rural Affairs, Xinjiang Key Laboratory of Agricultural Bio-Safety, Urumqi 830091, China; hewei8299@163.com (W.H.); wyg1176296665@outlook.com (Y.W.); shzutangsq@163.com (S.T.); lf576263465@163.com (W.L.); huangxin0924@126.com (X.H.); junhuiqzhou@163.com (J.Z.); kczx268@126.com (X.Z.); 2 College of Agriculture, Xinjiang Agricultural University, Urumqi 830091, China

**Keywords:** *Bacillus velezensis*, *Phelipanche aegyptiaca*, amino acids, synergistic effect

## Abstract

*Phelipanche aegyptiaca* is a root parasitic weed that causes severe yield losses in tomato production. Current control methods are constrained by limited efficacy and environmental concerns. Although biocontrol microbes and amino acids have each been reported to suppress broomrape parasitism individually, their synergistic effects and underlying molecular mechanisms remain largely unexplored. This study evaluated the biocontrol performance of *Bacillus velezensis* strain N35, applied alone or in combination with five amino acids (methionine, isoleucine, valine, histidine, and proline), against *P. aegyptiaca* parasitism in tomato using pot experiments coupled with transcriptomic profiling of host roots. Both individual and combined treatments significantly reduced the number and fresh weight of *P. aegyptiaca* parasitic tubercles. Notably, the combinations of methionine + N35 and isoleucine + N35 achieved near-complete suppression of parasitism. Transcriptomic analysis revealed extensive reprogramming of gene expression in tomato roots, with significant enrichment in pathways associated with plant hormone signal transduction, MAPK signaling, phenylpropanoid biosynthesis, and carotenoid biosynthesis. The synergistic treatments coordinately activated ethylene, jasmonic acid, and salicylic acid-mediated signaling, while suppressing auxin and abscisic acid signaling. Moreover, key strigolactone biosynthesis genes (*CCD7* and *CCD8*) were strongly downregulated, and specific genes involved in the biosynthesis of defense-related secondary metabolites were selectively upregulated. Collectively, these findings demonstrate a pronounced synergy between *B. velezensis* N35 and specific amino acids in suppressing *P. aegyptiaca* parasitism. This enhanced host resistance is achieved through the coordinated reprogramming of hormonal and metabolic networks, particularly via interference with strigolactone-mediated germination signal secretion. This study provides a theoretical basis for the development of microbe–metabolite synergistic strategies as sustainable and environmentally benign alternatives for broomrape management.

## 1. Introduction

The parasitic weed *Phelipanche aegyptiaca* severely threatens tomato production in arid and semi-arid regions [1]. By attaching to host roots and extracting water and nutrients, it causes substantial yield losses [2,3]. For instance, in tomato fields, yield losses due to *P. aegyptiaca* can reach as high as 65–70%, and even up to 80% in severe infestations [4]. Controlling broomrape is challenging due to its subterranean parasitism, metabolic integration with host plants, and persistent seed bank [5,6]. Current control strategies—such as cultural practices and chemical herbicides—have shown limited efficacy and raise environmental concerns [1,5,7]. Hence, the development of sustainable and eco-friendly alternatives is imperative.

Due to the advantages of environmental friendliness, high specificity, and good sustainability, microbial control has become a research hotspot in the green management of *Orobanche*. In recent years, remarkable progress has been made in the screening of biocontrol microbial resources, mechanism analysis, and field application at home and abroad [8,9,10,11]. A variety of bacteria, fungi and actinomycetes have been confirmed to exhibit significant inhibitory effects on *Orobanche* [12,13,14]. Among them, Iasur et al. reported that *Pseudomonas aeruginosa* PhelS10 could interfere with broomrape seed germination and haustorium development through biofilm formation, significantly reducing its parasitic success rate [15]. In addition, *Bacillus atrophaeus*, *Bacillus subtilis*, and *Azospirillum brasilense* have also been verified to exert significant inhibitory effects on *Orobanche aegyptiaca* and *Orobanche cumana* [16]. The biocontrol mechanisms mainly include secreting metabolites to inhibit seed germination [17], inducing suicidal germination of *Orobanche* in the absence of hosts [18], directly infecting parasitic plants leading to disease and death [19], regulating rhizosphere microecology, and enhancing the anti-parasitic ability of host crops [10].

Amino acids represent another innovative approach to broomrape control, primarily by disrupting crucial developmental processes such as germination [20,21,22]. Research indicates that certain amino acids can inhibit the early development of broomrape without harming the host plant. For example, Vurro et al. reported that the application of methionine and histidine in the tomato rhizosphere could inhibit the parasitism of *Orobanche ramosa* [20]. In addition, amino acids can induce inhibitory effects on plant growth by feedback inhibition of metabolic pathways, with the inhibition patterns being dependent on the plant species and developmental stage [22]. Studies have explored the use of amino acids to inhibit germination or deplete the soil seed bank of *Phelipanche ramose* [21]. This approach targets the weed before it can establish a parasitic connection with the host, thereby preventing infestation. The effectiveness of amino acid treatments against *O. ramosa* suggests their potential as novel compounds with higher herbicidal activity compared to conventional methods [20]. While the specific mechanisms often involve interfering with metabolic pathways, the precise amino acids and their optimal concentrations for broomrape control are subjects of ongoing investigation. Certain amino acids have been investigated for their ability to control *Orobanche minor* parasitism in red clover, with inhibitory efficacy varying significantly depending on both the chemical identity of the amino acid and the developmental stage of the parasite [22]. Amino acids can inhibit the germination and early development of broomrape by disrupting critical metabolic pathways without harming the host plant, offering an innovative and effective approach for controlling this parasitic weed. Notably, the synergistic strategy of microbial agents combined with low-risk chemicals or amino acids is becoming an important direction for the green control of broomrape.

Members of the genus *Bacillus* spp. produce a variety of antimicrobial substances and form highly stress-resistant endospores, resulting in their wide range of applications. Among them, the *B. velezensis* group serves as key biocontrol microorganisms in agriculture, exhibiting significant inhibitory effects against various plant pathogens and parasitic nematodes. Previously, our laboratory isolated and screened a strain of *B. velezensis* N35 with inhibitory activity against *P. aegyptiaca* from tomato rhizosphere soil. This study systematically investigated the synergistic effect of *B. velezensis* strain N35 in combination with specific amino acids on *P. aegyptiaca* parasitism in tomato plants. Utilizing a combination of pot trials and transcriptome sequencing, we comprehensively assessed alterations in parasitic parameters, plant physiological growth, and genome-wide transcriptional profiles. The objectives of this work were threefold: (1) to evaluate the biocontrol efficacy of individual and combined treatments; (2) to delineate the key metabolic and signaling pathways that were transcriptionally reprogrammed in response to these treatments; and (3) to elucidate the molecular mechanisms underlying the synergistic enhancement in host resistance mediated by amino acids and N35. Our results provide novel insights that contribute to the development of innovative and sustainable strategies for the management of root parasitic weeds.

## 2. Results

### 2.1. Efficacy of B. velezensis Strain N35 Against P. aegyptiaca

As shown in Table 1, different treatments had a significant effect (denoted by letter markers, *p* < 0.05) on both the number of parasites and the fresh weight of *P. aegyptiaca*. Specifically, compared to the water control group, the *B. velezensis* N35 treatment demonstrated significant control efficacy. Both the number of parasites (1.4 ± 0.54) and the fresh weight (0.86 ± 0.13 g) in the N35 treatment group were significantly lower than those in the control group (parasite count: 6.8 ± 1.09; fresh weight: 3.24 ± 0.39 g), indicating that the N35 strain effectively suppresses the parasitism and growth of *P. aegyptiaca*.

Notably, the Nutrient Broth (NB) medium alone also exhibited a certain inhibitory effect, with significantly lower parasite counts (4.8 ± 0.64) and fresh weight (1.96 ± 0.55 g) than the control. However, its efficacy was significantly weaker than that of the N35 treatment. This result suggests that the metabolites and/or the N35 strain itself play a primary role in suppressing *P. aegyptiaca*, rather than merely the nutritional components of the culture medium.

### 2.2. Effects of Amino Acids on P. aegyptiaca Infestation in Tomato Plants

**Figure 1:**
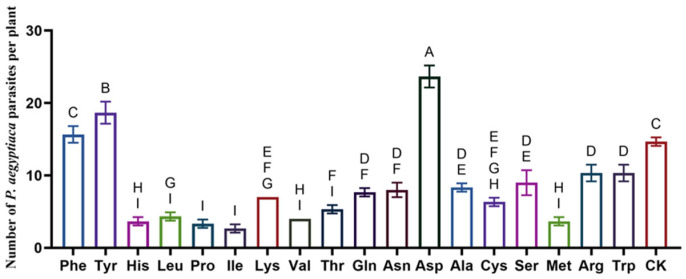
Effects of different amino acid treatments on the parasitism of *P. aegyptiaca* in tomato plants. Data are presented as mean ± SD (*n* = 9). Different letters indicate significant differences between treatments (Tukey’s HSD test, *p* < 0.05).

Figure 1 shows significant variation in the number of *P. aegyptiaca* parasitic tubercles across amino acid treatments. Aspartic acid (Asp) resulted in the highest number of parasitic tubercles, with a mean of 23.7 tubercles per plant—significantly exceeding all other treatments (*p* < 0.05). Tyrosine (Tyr) and phenylalanine (Phe) ranked second and third, with 18.7 and 16.3 tubercles per plant, respectively. The control (CK) exhibited 14.7 tubercles per plant, while isoleucine (Ile) suppressed nodulation most strongly, with only 2.7 tubercles per plant. Proline (Pro), histidine (His), methionine (Met), and valine (Val) also markedly inhibited nodulation, with means of 3.3, 3.7, 3.7, and 4.0 tubercles per plant, respectively.

### 2.3. Synergistic Effect of B. velezensis N35 Combined with Amino Acids on Controlling P. Aegyptiacain in Tomato

**Figure 2:**
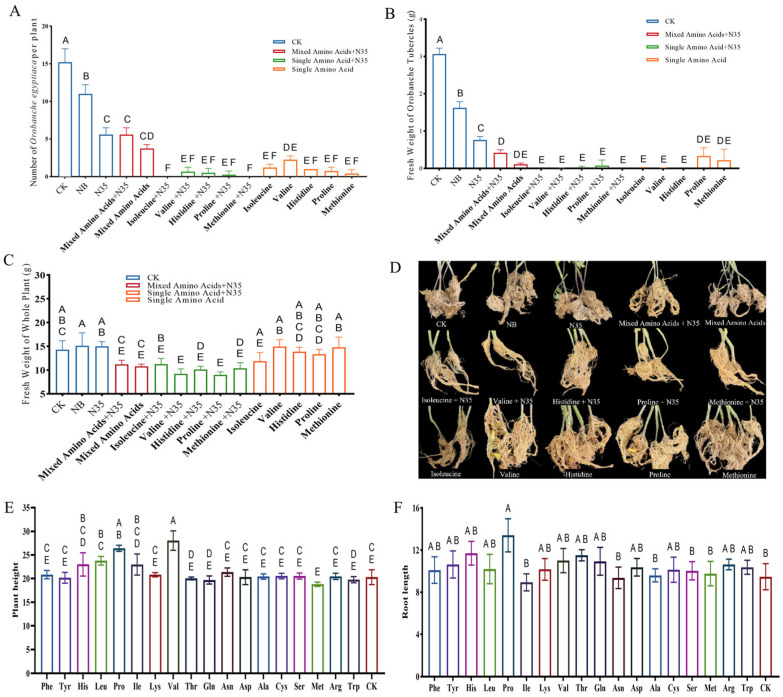
Effects of amino acid and N35 treatments on *P. aegyptiaca* parasitism and host plant growth. Data are presented as mean ± SD (*n* = 9 biological replicates). (**A**) Number of *P. aegyptiaca* parasites per plant; (**B**) fresh weight of *P. aegyptiaca* tubercles; (**C**) fresh weight of host (tomato) plants; (**D**) representative images of host plants and root systems under different treatments; (**E**) height of tomato; (**F**) root length of tomato. Different letters indicate significant differences between treatments (Tukey’s HSD test, *p* < 0.05).

The experimental results indicated that all treatments significantly reduced both the number of parasitic *P. aegyptiaca* and their fresh weight on tomato plants compared to the water control (CK, 14.7 tubercles per plant) (Figure 2D). Among them, the methionine (Met) + N35 and isoleucine (Ile) + N35 treatments demonstrated the strongest inhibitory effects, completely suppressing the parasitism of *P. aegyptiaca* (Figure 2A), with corresponding fresh weights reduced to zero (Figure 2B).

In terms of parasite count, all single amino acid treatments and their combinations with N35 showed significant control efficacy (Figure 2A). Notably, the combined application of amino acids with N35 exhibited a synergistic effect in reducing the number of parasites compared to the corresponding single amino acid treatments (Figure 2A), suggesting that the co-application of the N35 strain with specific amino acids can significantly enhance the control of *P. aegyptiaca*.

Regarding the biomass of *P. aegyptiaca*, the fresh weight under all treatments was significantly lower than that of the CK (*p* < 0.05), with reductions ranging from 75.16% to 99.78% (Figure 2B). It is noteworthy that, apart from CK, the fresh weight of tubers in the NB (Nutrient Broth) treatment group was also significantly higher than that in other biological treatment groups (Figure 2B). This indicates that the basal medium promoted the growth of *P. aegyptiaca* to some extent, highlighting the importance of exogenous additives in regulating the parasitic process. Overall, the combined treatments of methionine and isoleucine with N35 were the most effective in suppressing both parasitism and biomass accumulation of *P. aegyptiaca*.

As shown in Figure 2C, different treatments influenced the overall fresh weight of tomato plants. Compared with the control (CK, approximately 15.0 g), most treatment groups showed a reduction in plant fresh weight. In particular, certain combined treatments of amino acids with N35—such as valine + N35, histidine + N35, and proline + N35—exhibited a more pronounced decrease, with fresh weights falling within the range of 6.0 g–8.0 g. This suggests that, while strongly suppressing *P. aegyptiaca* parasitism, some combined treatments may have partially inhibited tomato plant growth, or may reflect dynamic changes in biomass allocation following the alleviation of parasitic stress. In contrast, the fresh weights, height and root length of plants in the NB and N35-alone treatment groups were similar to or slightly higher than those of the CK, indicating that under the experimental conditions, microbial inoculation alone or the basic nutrient supply had no significant adverse effect on tomato growth (Figure 2C–F).

### 2.4. Transcriptomic Analysis of Tomato Roots Treated with N35 and Amino Acid Analysis of Differentially Expressed Genes

#### 2.4.1. Analysis of Differentially Expressed Genes

**Figure 3:**
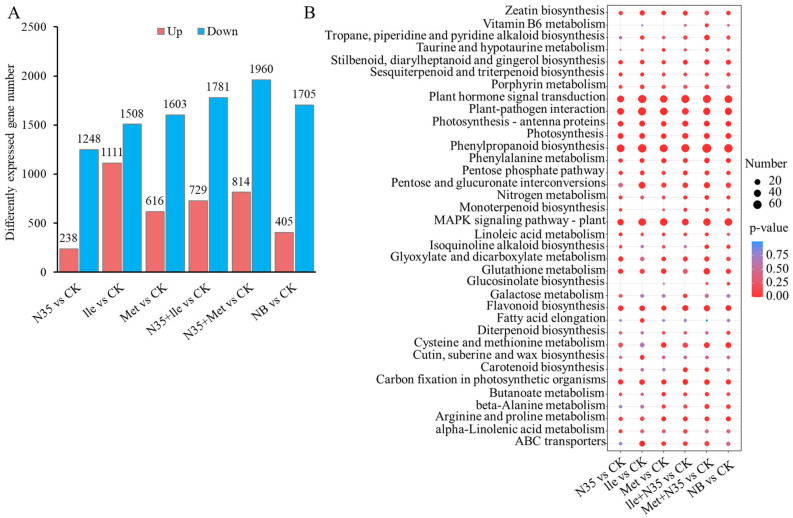
Analysis of differentially expressed genes and KEGG enrichment in tomato roots. (**A**) Number of differentially expressed genes in tomato roots in response to amino acid treatments; (**B**) KEGG pathway enrichment analysis of the top 20 differentially expressed genes in tomato roots.

Transcriptomic analysis revealed that, compared with the control group (CK), the numbers of upregulated genes in the biocontrol strain N35 (N35), isoleucine (Ile), methionine (Met), N35 + Ile, N35 + Met, and NB treatment groups were 238, 1111, 616, 729, 814, and 405, respectively. The corresponding numbers of downregulated genes were 1248, 1508, 1603, 1781, 1960, and 1705 (Figure 3A).

The KEGG pathway enrichment analysis of differentially expressed genes in tomato roots is shown in Figure 3B. The results indicated that all treatments significantly influenced the metabolic and signaling pathways of tomato roots, particularly those related to secondary metabolism, hormone signaling, and stress response.

Compared with single treatments, the combined treatments of N35 with amino acids (Ile + N35 and Met + N35) induced more extensive and significant changes in gene expression across most pathways (Figure 3B). For instance, the “Plant hormone signal transduction” and “MAPK signaling pathway–plant” pathways were significantly enriched in the combined treatment groups, suggesting a synergistic role of N35 and amino acids in regulating plant defense and stress adaptation mechanisms. Additionally, in pathways such as “Phenylpropanoid biosynthesis” and “Flavonoid biosynthesis,” all treatments except the NB control showed significant enrichment, with the combined treatments exhibiting the highest number of enriched genes and the most significant *p* values. This implies that the combination of N35 and specific amino acids may cooperatively enhance the synthesis of defense-related secondary metabolites.

Treatments involving Met, Ile, and their combinations also significantly affected the “Cysteine and methionine metabolism” and “alpha Linolenic acid metabolism” pathways, indicating a potential regulatory role of these amino acids in sulfur metabolism and jasmonic acid-mediated defense responses (Figure 3B). Notably, the “ABC transporters” pathway was significantly enriched across all treatments, with the Met + N35 group showing the highest number of enriched genes, suggesting an active regulation of membrane transport and detoxification processes (Figure 3B).

In contrast, the NB control group exhibited limited and mostly non-significant alterations across the pathways, further supporting that the observed transcriptional responses were primarily driven by the N35 and amino acid treatments (Figure 3B). In summary, the combined application of *B. velezensis* N35 with specific amino acids broadly reprograms the metabolic and signaling networks in tomato roots, particularly enhancing the transcriptional regulation of defense-related, hormone signaling, and secondary metabolic pathways.

#### 2.4.2. Integrated Analysis of Tomato Root Transcriptomics and *P. aegyptiaca* Parasitism Effect

**Figure 4:**
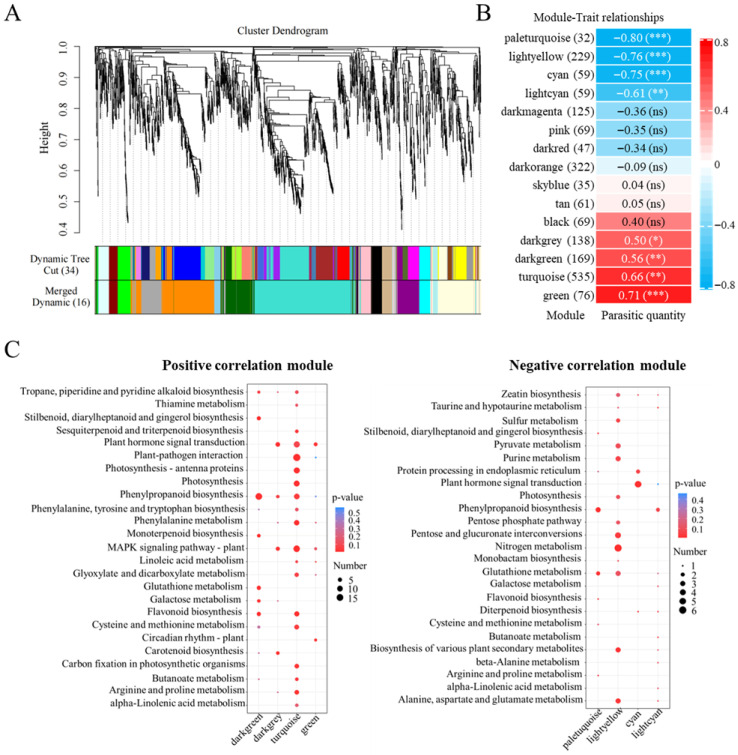
Weighted gene co-expression network analysis (WGCNA) of tomato roots in response to *P. aegyptiaca* parasitism. (**A**) Gene clustering dendrogram and module identification; (**B**) module–trait correlation heatmap depicting associations between co-expression modules and parasitic quantity; (**C**) top 20 KEGG pathway enrichment analysis for positively and negatively correlated modules. * *p* < 0.05, ** *p* < 0.01, *** *p* < 0.001, ns: not significant.

Clustering analysis of tomato root transcriptomic data under different treatment conditions and phenotypic data of *P. aegyptiaca* parasitism quantity identified a total of 15 co-expression modules. Among these, the gene expression levels in the lightyellow, pink, cyan, paleturquoise, darkred, and lightcyan modules were negatively correlated with the number of parasitic plants, whereas the turquoise, black, darkgrey, darkgreen, and green modules showed a positive correlation (Figure 4A,B). KEGG pathway enrichment analysis revealed that the negatively correlated modules were significantly enriched in pathways related to plant hormone signal transduction, nitrogen metabolism, and glutathione metabolism. In contrast, the positively correlated modules were significantly enriched in pathways including plant hormone signal transduction, phenylpropanoid biosynthesis, MAPK signaling pathway, and carotenoid biosynthesis (Figure 4C).

#### 2.4.3. Changes in Carotenoid Biosynthesis Pathway in Tomato Roots Treated with Amino Acids and N35

**Figure 5:**
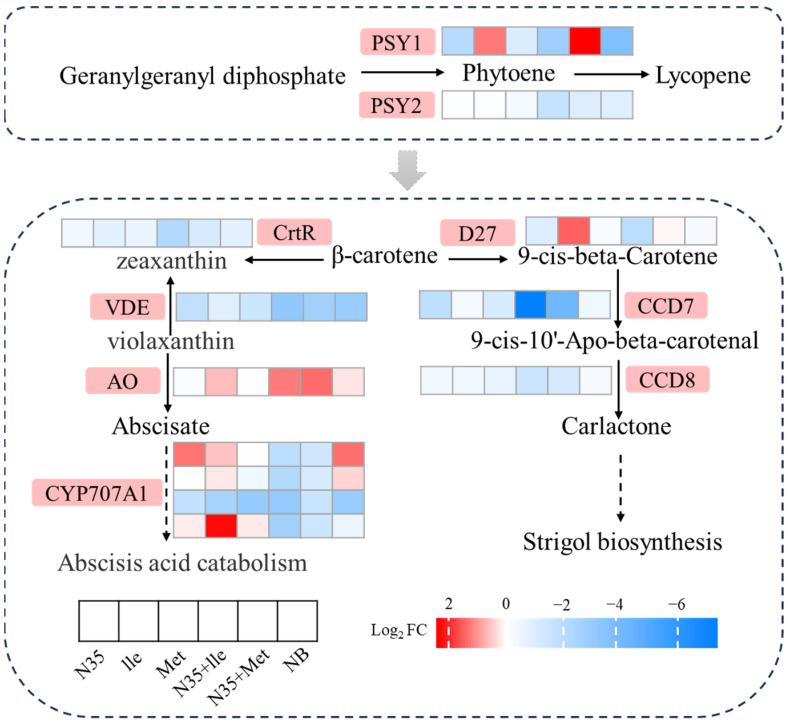
Alterations in the carotenoid biosynthesis pathway under different treatments.

Analysis of genes related to the carotenoid biosynthesis pathway revealed significant transcriptional reprogramming. Combined treatments with the biocontrol strain N35 and amino acids (N35 + Ile, N35 + Met) triggered this reprogramming, which was predominantly characterized by downregulation. Within the abscisic acid (ABA) biosynthetic branch of the carotenoid pathway, four ABA 8ʹ-hydroxylase genes (*CYP707A1*, *CYP707A2*, *CYP707A4*, and one *CYP707A2* like) were markedly downregulated in both the Ile + N35 and Met + N35 groups. Analysis of the strigolactone biosynthesis branch indicated that its key genes were strongly suppressed under the combined treatments. The carotenoid cleavage dioxygenase gene *CCD7* exhibited the most pronounced downregulation, with log_2_FC values of −7.3 in the N35 + Ile group and −4.1 in the N35 + Met group. The downstream gene *CCD8* and the upstream β-carotene isomerase gene *D27* were also significantly downregulated in the corresponding treatment group, with log_2_FC values of −1.5 (≈2.8 fold decrease) and −1.9 (≈3.7 fold decrease), respectively (Figure 5).

#### 2.4.4. Effects of Different Treatments on Defense- and Growth-Related Hormone Signaling Pathways in Tomato Roots

**Figure 6:**
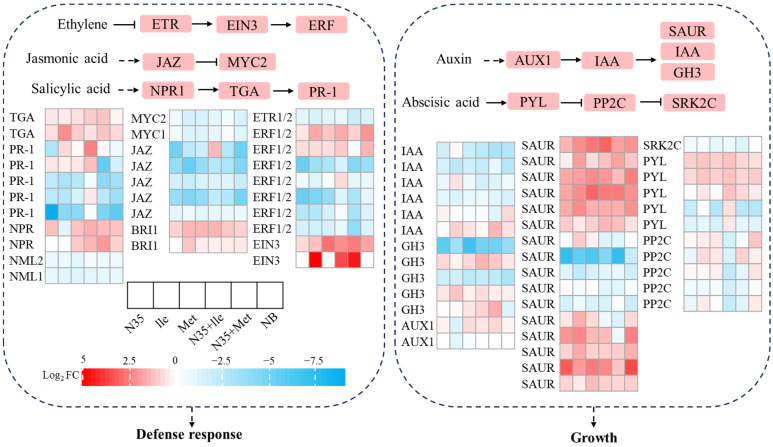
Regulation of plant hormone signaling pathways in tomato roots treated with amino acids and N35.

In the defense-related hormone signaling pathways (left panel of Figure 6), the expression of key receptor genes in ethylene signaling, such as *ETR1/2*, and their downstream transcription factor *EIN3* was upregulated in most treatments. The negative regulator *JAZ* in the jasmonic acid pathway was significantly downregulated under N35 combined treatments (N35 + Ile, N35 + Met), while its target transcription factor *MYC2* was downregulated. The key regulatory factor *NPR1* and its interacting partner *TGA* in the salicylic acid pathway also showed enhanced expression in some treatments, particularly with N35 alone. These results suggest that the biocontrol agent N35, especially in combination with amino acids, synergistically activates ethylene, jasmonic acid, and salicylic acid signaling pathways, thereby enhancing the basal immune response in tomato roots.

In the growth-related hormone pathways (right panel of Figure 6), the expression of early response genes in auxin signaling, such as *SAUR*, and the auxin influx carrier *AUX1* was suppressed in most treatments, with particularly pronounced downregulation in the N35 + Met treatment. The receptor gene *PYL* and its downstream regulatory modules in the abscisic acid pathway were generally downregulated in the combined treatment groups, indicating that abscisic acid-mediated stress responses were partially suppressed. These findings suggest that while activating defense responses, the root system may temporarily delay growth processes by inhibiting auxin and abscisic acid signaling pathways, thereby reallocating resources between “growth” and “defense”.

#### 2.4.5. Changes in the Phenylpropanoid Pathway of Tomato Roots in Response to Treatment with Amino Acids and *B. velezensis* N35

**Figure 7:**
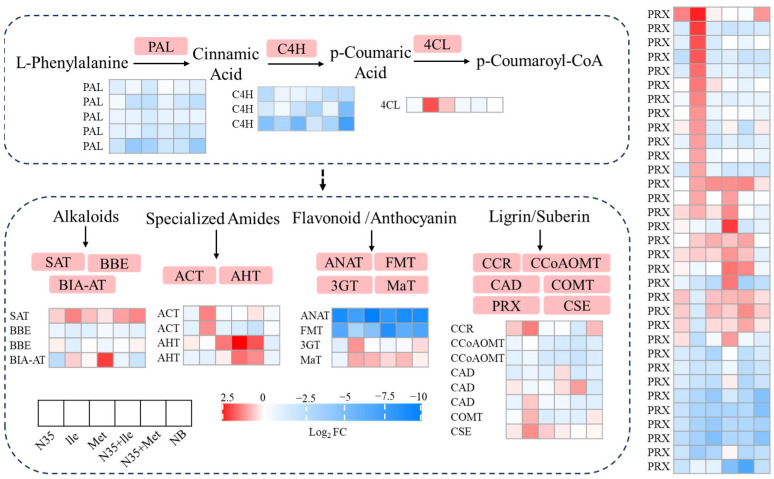
Effects of amino acid and *B. velezensis* N35 treatments on the phenylpropanoid metabolic pathway in tomato roots.

Synergistic treatment with *B. velezensis* N35 and amino acids (Ile or Met) significantly altered phenylpropanoid metabolism in tomato roots. The expression of *PAL*, which encodes the initial enzyme of the phenylpropanoid pathway, was generally and markedly downregulated. Key genes involved in the lignin-specific branch were also suppressed, including cinnamoyl-CoA reductase (*CCR*) (log_2_FC = −1.72 in treatment E) and several anionic peroxidase genes (*PRX*) related to lignin formation. While most *PRX* genes were downregulated, a few individual *PRX* genes were upregulated in the Ile + N35 and Met + N35 treatments. In contrast, genes encoding enzymes for the synthesis of specific defense compounds were selectively induced. Two agmatine hydroxycinnamoyltransferase genes (*AHT*) were strongly and highly upregulated under combined Ile + N35 and Met + N35 treatments. Additionally, the stemmadenine O-acetyltransferase (*SAT*) gene, involved in alkaloid biosynthesis, was significantly upregulated in the Met + N35 treatment (log_2_FC = 2.19 and 2.74, respectively) (Figure 7).

## 3. Discussion

This study confirms that the biocontrol agent *B. velezensis* N35, in combination with specific amino acids—particularly methionine and isoleucine—exhibits a significant synergistic effect in suppressing the parasitism of *P. aegyptiaca*. This synergistic interaction was not only phenotypically evident through the near-complete inhibition of parasitism but also reflected at the transcriptomic level by profound and coordinated reprogramming of the defense network in tomato roots. These findings align with previous reports on the ability of individual microorganisms or amino acids to modulate plant resistance [23,24,25] and further expand the concept of “microbe–metabolite” synergistic regulation.

The modulation of plant hormone networks by the combined treatments appears particularly crucial. The results demonstrate that N35 and amino acids cooperatively activated ethylene, jasmonic acid, and salicylic acid signaling pathways, which are central to systemic plant immunity [26,27,28,29]. The current data reveal a critical distinction: the addition of methionine—a precursor in ethylene biosynthesis—appears to potentiate the JA signaling cascade beyond the threshold achievable by N35 alone. Specifically, the pronounced downregulation of *JAZ* and *MYC2* suggests a “gating” mechanism. We hypothesize that the methionine-derived ethylene burst sensitizes the root tissue, allowing for a more rapid and robust transcriptional response to the microbe-associated molecular patterns (MAMPs) presented by *B. velezensis* N35. This cross-talk alleviates the negative feedback loops that typically constrain JA responses, thereby locking the root into a heightened defensive state. Conversely, the observed suppression of auxin and abscisic acid (ABA) signaling aligns with the classic “growth–defense trade-off” conceptual framework. However, our transcriptomic data offer a nuanced view: this is not a passive consequence of resource depletion, but an active redirection of transcriptional machinery away from cell elongation and toward secondary metabolite biosynthesis. The moderate reduction in tomato fresh weight observed under specific amino acid combinations thus reflects the energetic cost of this sustained, primed immune status rather than direct phytotoxicity.

The most striking mechanistic insight is the strong suppression of the strigolactone biosynthetic pathway by the synergistic treatments. Strigolactones serve as key signaling molecules that stimulate seed germination of parasitic weeds such as *P. aegyptiaca* [30,31]. The severe downregulation of *CCD7* and *CCD8*—genes encoding carotenoid cleavage dioxygenases essential for SL production. In the context of parasitic weed management, the host root exudate profile functions as a “chemical beacon”. By silencing this beacon through the suppression of *CCD7* and *CCD8*, the combined treatment effectively renders the host root “invisible” to the germinating seeds of *P. aegyptiaca*. This finding has broader implications than previously appreciated: it suggests that the *B. velezensis* N35–amino acid combination does not merely fortify the cell wall against penetration but actively disrupts the obligate signaling dialog between host and parasite. This pre-infection defense mechanism is likely more evolutionarily stable than post-attachment hypersensitive responses, as it exerts minimal selective pressure on the parasite population to evolve virulence factors.

Furthermore, the differential expression of phenylpropanoid pathway genes further underscores the sophistication of the host response. Reprogramming of the phenylpropanoid metabolic pathway suggests that the combined treatments may promote the synthesis of specific defense compounds, such as hydroxycinnamic acid amides or related alkaloids, rather than inducing generalized lignification [32,33]. This targeted approach is metabolically efficient. It conserves carbon skeletons that would otherwise be deposited as structural lignin, while simultaneously generating low-molecular-weight compounds with potent antimicrobial or signaling-modulatory properties. Notably, the NB group exhibited a certain promotive effect on parasitism, underscoring the complex influence of rhizosphere nutritional conditions on parasitic interactions and, in contrast, emphasizing the specific functional role of the biocontrol agent and its metabolites. This serves as an important reminder that merely enriching the rhizosphere with carbon and nitrogen can inadvertently increase host susceptibility by providing the parasite with metabolic cues or by diluting specific microbial signals.

In addition, accumulating evidence has demonstrated that biocontrol microorganisms can effectively suppress parasitic weeds by reshaping the structure, diversity, and function of the soil microbial community, especially in the rhizosphere [10,34]. Beneficial strains can optimize the rhizosphere microecosystem via niche competition, metabolite cross-talk, signal interference, and enrichment of antagonistic microbes, thereby forming a long-term soil-borne barrier that inhibits the germination and infection of *P. aegyptiaca* [35]. In the present study, the outstanding control efficacy of methionine + N35 against *P. aegyptiaca* has been mainly attributed to the induced resistance in tomato plants, including hormonal reprogramming, immune activation, and downregulation of strigolactone biosynthesis. Nevertheless, it remains unclear whether this synergistic combination also functions by modulating the rhizosphere soil microbial community. We cannot rule out the possibility that methionine + N35 may cooperatively shape the composition of bacteria and fungi, enhance the relative abundance of beneficial taxa, reduce the growth-promoting environment for broomrape seeds, and thus contribute to the suppression of parasitism in an indirect but sustainable manner. Therefore, further investigations combining microbiome sequencing, metabolomics, and synthetic microbial communities are urgently needed to clarify whether the regulation of soil microecology serves as an additional mechanism underlying the synergistic effect of *B. velezensis* N35 and methionine.

Admittedly, the observation that some amino acid–N35 combinations slightly inhibited tomato growth indicates the need for further optimization of formulation parameters—such as amino acid type, concentration, and application method—to achieve an optimal balance between pest suppression and plant growth promotion. Moreover, the stability of the synergistic effect under field conditions and its long-term impact on soil microbiomes remain to be validated in subsequent studies.

## 4. Materials and Methods

### 4.1. Experimental Materials

*B. velezensis* strain N35 was isolated from the rhizosphere soil of *P. aegyptiaca* in a processing tomato field. *P. aegyptiaca* seeds were collected from naturally matured plants in a processing tomato field located in Jimsar County, Xinjiang Uygur Autonomous Region, China. An amount of 10 g of soil sample was weighed, dispersed by shaking in sterile physiological saline, and subjected to gradient dilution. An amount of 100 μL of dilutions ranging from 10^−3^ to 10^−7^ was spread onto NA plates, followed by inverted incubation at 28 °C for 1–2 d. Single colonies with distinct morphological characteristics were picked and purified three times successively by the streak plate method to obtain pure cultures, which were then preserved in LB medium containing 30% sterile glycerol at −20 °C. Total genomic DNA of the strain was extracted, and the 16S rRNA and gyrB genes were amplified by PCR and subjected to bidirectional sequencing. BLAST (v2.17.0) homology analysis against the NCBI database showed that the 16S rRNA gene (GenBank accession no.: PV450598.1) and gyrB gene (GenBank accession no.: PV554280.1) of strain N35 shared 99% similarity with the corresponding sequences of *Bacillus velezensis* ZF514 (GenBank accession nos.: OR899803.1 and OR909914.1). Based on 16S rDNA and gyrB gene sequencing, combined with colony morphology, physiological and biochemical characteristics, and molecular identification results, strain N35 was identified as *B*. *velezensis*.

The tomato cultivar used was *Xinhong 55*, kindly provided by the Institute of Fruits and Vegetables, Xinjiang Academy of Agricultural Sciences. The synthetic strigolactone analog GR24 was purchased from Macklin Biochemical Co., Ltd. (Shanghai, China). The amino acids—methionine (Met), threonine (Thr), leucine (Leu), isoleucine (Ile), histidine (His), proline (Pro), serine (Ser), aspartic acid (Asp), asparagine (Asn), alanine (Ala), phenylalanine (Phe), valine (Val), tyrosine (Tyr), glutamine (Gln), cysteine (Cys), arginine (Arg), lysine (Lys), and tryptophan (Trp)—were also obtained from Macklin Biochemical Co., Ltd. (Shanghai, China).

### 4.2. Pot Experiment for Control of P. aegyptiaca by B. velezensis N35

#### 4.2.1. Sterilization of *P. aegyptiaca* Seeds

*P. aegyptiaca* seeds were surface sterilized by immersion in 75% (*v*/*v*) ethanol for 1.5 min, followed by treatment with 1% (*v*/*v*) sodium hypochlorite for 12 min. After sterilization, the seeds were spread on sterile filter paper and air-dried in a laminar flow cabinet for subsequent use.

#### 4.2.2. Preparation of Bacterial Fermentation Broth

*B. velezensis* strain N35 was activated by streaking onto nutrient agar (NA) plates and incubating at 28 °C for 48 h. A single colony was selected and sub-cultured twice successively. The activated strain was then transferred using a sterile pipette tip into a 300 mL Erlenmeyer flask containing 50 mL of Nutrient Broth (NB) medium. The culture was incubated at 30 °C with shaking at 180 rpm for 48 h to obtain the working bacterial suspension.

#### 4.2.3. Pot Experimental Design and Sampling Protocol

The experiment comprised three treatments: *B. velezensis* N35, NB medium, and a blank control (water). Each treatment was replicated nine times, with one pot per replicate. Plastic pots (9 cm in diameter) were used and filled to approximately one-third of their volume with soil. A mixture of 100 mg of *P. aegyptiaca* seeds and 20 g of soil was evenly spread into each pot. Tomato seedlings at the 5–6 true-leaf stage were transplanted into the pots and placed in a controlled-environment growth chamber. Seven days after transplanting, 50 mL of a bacterial suspension with a concentration of 1 × 10^8^ CFU·mL^−1^ was applied to each pot in the N35 treatment group. The same volume of NB medium was applied to the NB treatment group, and an equal volume of water was applied to the control group. Forty days after transplanting, all tomato plants were carefully uprooted. The roots were gently washed to remove adhering soil, after which the number of *P. aegyptiaca* tubercles attached to the roots was counted, and their fresh weight was measured.

#### 4.2.4. Pot Experiment on the Control of *P. aegyptiaca* with Various Amino Acids

Each of the 18 amino acids was prepared as an aqueous solution at a concentration of 0.5 g/L. The experiment consisted of 19 treatments, including 18 individual amino acid treatments and a blank control (water). Each amino acid treatment group was irrigated with 100 mL of the corresponding amino acid solution, while the control group received an equal volume of distilled water. All other procedures in the pot experiment followed the methods described in Section 4.2.1, Section 4.2.2 and Section 4.2.3.

#### 4.2.5. Pot Experiment on the Control of *P. aegyptiaca* with Amino Acids Combined with *B. velezensis* N35

Five individual amino acid solutions were prepared at a mass fraction of 0.4%. Each amino acid was then separately dissolved in an N35 bacterial suspension at the same mass fraction (0.4%). Additionally, a mixed amino acid solution and an amino acid-bacterial suspension were prepared by dissolving the five amino acids (total concentration of 0.08%, *w*/*v*) in 1000 mL of sterile water and 1000 mL of the N35 suspension, respectively.

Fifteen treatments were established: methionine (Met), proline (Pro), histidine (His), valine (Val), isoleucine (Ile), mixed amino acids, Met + N35, Pro + N35, His + N35, Val + N35, Ile + N35, mixed amino acids + N35, N35 alone, Nutrient Broth (NB) medium, and a blank control (distilled water). Each treatment was replicated ten times, with one pot per replicate.

The surface sterilization of *P. aegyptiaca* seeds and the preparation of the *B. velezensis* N35 suspension followed the procedures described in Section 4.2.1 and Section 4.2.2. Plastic pots (9 cm in diameter) were filled to approximately one third of their volume with soil. A mixture of 100 mg of *P. aegyptiaca* seeds and 20 g of soil was evenly spread into the pots.

Tomato seedlings at the 5–6 true leaf stage were removed from seedling trays, immersed in the respective treatment solutions for 1 h, and then transplanted into the prepared pots. All pots were placed in a controlled environment growth chamber and subjected to routine watering and fertilization.

Forty days after transplanting, all plants were carefully uprooted. The roots were gently washed to remove adhering soil, after which the number of *P. aegyptiaca* tubercles, their fresh weight, and the fresh weight of the tomato plants were recorded.

### 4.3. Transcriptome Analysis

Based on the experimental results in Section 4.2, this study selected 5 treatments (N35, Ile, Met, N35 + Ile, N35 + Met) that exhibited significant inhibitory effects on *Orobanche* to treat tomato plants, with the treatment method consistent with that described in Section 4.2.5. Tomato plant samples were collected at 20 days after transplanting. Root systems were rinsed with clean water to remove adhering soil, gently blotted dry with absorbent paper, and approximately 0.5 g of fresh root tissue was excised and transferred into 15 mL centrifuge tubes. Samples were immediately flash-frozen in liquid nitrogen and stored at −80 °C. Each treatment was performed in three biological replicates. Transcriptome sequencing was performed by Shanghai OE Biotech Co., Ltd. (Shanghai, China). Total RNA was extracted from samples using the Trizol reagent. RNA purity and concentration were assessed spectrophotometrically using a NanoDrop 2000 instrument (Thermo Fisher Scientific, Waltham, MA, USA). RNA integrity was verified by capillary electrophoresis on an Agilent 2100 Bioanalyzer (Agilent Technologies, Santa Clara, CA, USA). Strand-specific RNA-seq libraries were prepared using the VAHTS Universal V6 RNA-seq Library Prep Kit (Premixed Version) (Vazyme Biotech, Nanjing, China), with ribosomal RNA depletion performed prior to fragmentation for poly(A)-negative samples where applicable. Sequencing was conducted on an Illumina NovaSeq 6000 platform (Illumina, San Diego, CA, USA) to generate 150 bp paired-end reads. Raw sequencing reads (FASTQ format) were subjected to quality control and adapter trimming using fastp (v0.23.2), with low-quality bases (Q < 20), reads containing ambiguous ‘N’ bases (>1%), and reads shorter than 36 bp being discarded; the resulting high-quality clean reads were used for downstream analysis. Clean reads were aligned to the tomato reference genome using HISAT2 (v2.2.1). Gene-level expression was quantified in both FPKM (Fragments Per Kilobase of transcript per Million mapped reads) and raw read counts; FPKM values facilitated cross-sample expression visualization and correlation analysis, whereas raw counts served as input for differential expression testing. Differential expression analysis was performed using DESeq2 (v1.36.0), applying the Wald test with Benjamini–Hochberg adjustment for multiple testing; genes exhibiting |log_2_(fold change)| ≥ 1 and adjusted *p*-value (q-value) < 0.05 were designated as differentially expressed genes (DEGs). Functional enrichment analysis of DEGs was carried out separately for Gene Ontology (GO) terms and KEGG pathways using the hypergeometric test, with significance defined as *p*-value < 0.05 after BH correction.

### 4.4. Statistical Analysis

Data are presented as the mean ± standard error (SD) and were analyzed by one-way analysis of variance (ANOVA). A completely randomized design (CRD) was used for the pot experiment. Statistical analysis was performed using SPSS 25.0. Significant differences among treatments were determined by the least significant difference (LSD) test, and multiple comparisons are indicated with lowercase letters.

## 5. Conclusions

**Figure 8:**
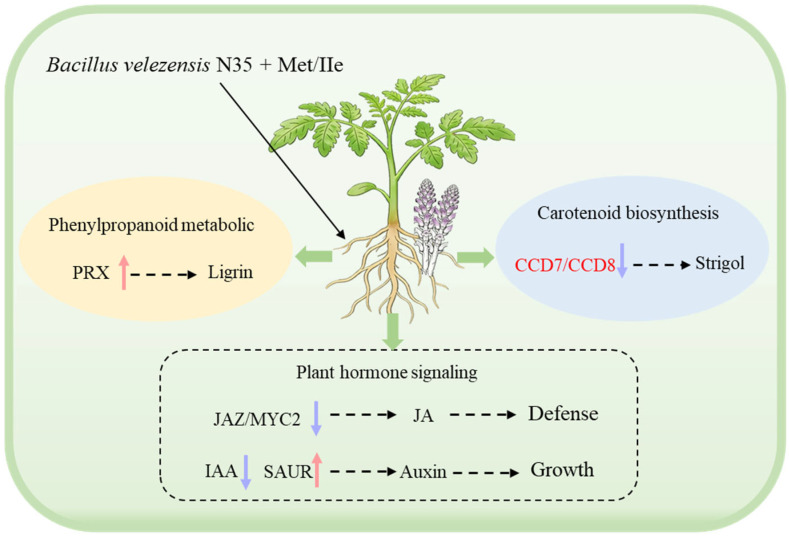
Schematic model of the synergistic defense mechanisms induced by *Bacillus velezensis* N35 and amino acids against *Phelipanche aegyptiaca* parasitism in tomato roots.

This study demonstrates that co-application of *B. velezensis* N35 with methionine or isoleucine achieves near-complete suppression of *P. aegyptiaca* parasitism on tomato, validating a synergistic “microbe–metabolite” strategy for parasitic weed control. Mechanistically, this enhanced resistance is mediated primarily through the remodeling of host hormonal networks and metabolic fluxes, notably the attenuation of strigolactone signaling, which intercepts parasitism at the germination stage. While the activation of these defenses was associated with a moderate growth–defense trade-off under certain treatments, the findings underscore the feasibility of this targeted approach (Figure 8). Future research should prioritize formulation optimization to mitigate growth impacts and validate efficacy and ecological safety under field conditions.

## Figures and Tables

**Table 1:** Effects of different treatments on parasitism and fresh weight of *P. aegyptiaca*.

Treatment	Tubercles Count	Tubercles FW (g)
N35	1.4 ± 0.54 c	0.86 ± 0.13 c
NB (Nutrient Broth)	4.8 ± 0.64 b	1.96 ± 0.55 b
Control (Water)	6.8 ± 1.09 a	3.24 ± 0.39 a

Values are presented as mean ± SD (*n* = 9). Different lowercase letters within the same column indicate significant differences among treatments according to Tukey’s HSD test (*p* < 0.05).

## Data Availability

All the data related to this project are presented here.

## Abbreviations

The following abbreviations are used in this manuscript:

NB	Nutrient Broth
NA	Nutrient agar (NA)
Met	Methionine
Thr	Threonine
Leu	Leucine
Ile	Isoleucine
His	Histidine
Pro	Proline
Ser	Serine
Asp	Aspartic acid
Asn	Asparagine
Ala	Alanine
Phe	Phenylalanine
Val	Valine
Tyr	Tyrosine
Gln	Glutamine
Cys	Cysteine
Arg	Arginine
Lys	Lysine
Trp	Tryptophan
